# Intelligence

**Published:** 1832-10

**Authors:** 


### INTELLIGENCE.

### KING'S COLLEGE, LONDON.

SEVERAL additional presents of books have lately been received; and the Council have voted, we hear, a further sum for the immediate purchase of standard works. We doubt not, when it is generally known that a reading room is opened, and a library of reference forming for the use of the students, that the collection will rapidly increase by donations from the friends and patrons of the institution.

Professor GREEN's introductory lecture will be given this day, October 1st, at three P.M.; Professor MAYO's, on Tuesday, Oct. 2d; and Professor BURNETT's, on Wednesday, Oct. 3d, at the same hour. The classical department will open on Monday, the 8th of October.

---

Mr. A. S. TAYLOR will commence his Lectures on Medical Jurisprudence, in the theatre of Guy's Hospital, on Tuesday, the 13th of October, at half-past three P.M. The course consists of four parts: the first comprising "Questions relative to the various forms of sudden or violent death;" the second, "Questions relating to the generative and parturient systems;" the third, "Questions relating to cases of a miscellaneous nature;" the fourth, "Questions relating to medical police."

---

*Medical Appointment.* Dr. J. WYATT CRANE has been elected physician to the St. George's and St. James's Dispensary, in the room of Dr. SOMERVILLE, resigned.

---

*Surgeon to Chelsea Hospital.* The valuable appointment of surgeon to Chelsea Hospital, which was held by Sir Everard Home, is to be conferred on Mr. KEATE.

---

*Sergeant Surgeon.* We understand that Mr. BRODIE is to be Sergeant-Surgeon to the King, in the room of Sir E. HOME. It is said his late Majesty made this appointment a matter of special request to his royal brother, then Duke of Clarence, when it was all but certain that he would be his immediate successor to the crown.—*Med. Gazette.*

---

### AN ACT FOR REGULATING SCHOOLS OF ANATOMY;
*Being the 2d and 3d of* William IV. cap. lxxv.

WHEREAS a knowledge of the causes and nature of sundry diseases which affect the body, and of the best methods of treating and curing such diseases, and of healing and repairing divers wounds and injuries to which the human frame is liable, cannot be acquired without the aid of anatomical examination: and whereas the legal supply of human bodies for such anatomical examination is insufficient fully to provide the means of such knowledge: and whereas, in order further to supply human bodies for such purposes, divers great and grievous crimes have been committed, and lately murder, for the single object of selling for such purposes the bodies of the persons so murdered; and whereas, therefore, it is highly expedient to give protection, under certain regulations, to the study and practice of anatomy, to prevent, as far as may be, such great and grievous crimes and murder as aforesaid; be it therefore enacted by the King's Most Excellent Majesty, by and with the advice and consent of the Lords spiritual and temporal, and Commons, in this present parliament assembled, and by the authority of the same, that it shall be lawful for his Majesty's principal secretary of state, for the time being, for the home department, in that part of the United Kingdom called Great Britain, and for the chief secretary for Ireland, in that part of the United Kingdom called Ireland, immediately on the passing of this Act, or so soon thereafter as may be required, to grant a licence to practise anatomy to any fellow or member of any college of physicians or surgeons, or to any graduate or licentiate in medicine, or to any person lawfully qualified to practise medicine in any part of the United Kingdom, or to any professor or teacher of anatomy, medicine, or surgery, or to any student attending any school of anatomy, on application from such party for such purpose, counter-signed by two of his Majesty's justices of the peace acting for the county, city, borough, or place, wherein such party resides, certifying that, to their knowledge or belief, such party so applying is about to carry on the practice of anatomy.

II. And be it enacted, that it shall be lawful for his Majesty's said principal secretary of state, or chief secretary, as the case may be, immediately on the passing of this Act, or as soon thereafter as may be necessary, to appoint respectively not fewer than three persons to be inspectors of places were anatomy is carried on, and at any time after such first appointment, to appoint, if they shall see fit, one or more other person or persons to be an inspector or inspectors as aforesaid; and every such inspector shall continue in office for one year, or until he be removed by the said secretary of state, or chief secretary, as the case may be, or until some other person shall be appointed in his place; and as often as any inspector appointed as aforesaid shall die, or shall be removed from his said office, or shall refuse or become unable to act, it shall be lawful for the said secretary of state, or chief secretary, as the case may be, to appoint another person to be inspector in his room.

III. And be it enacted, that it shall be lawful for the said secretary of state or chief secretary, as the case may be, to direct what district of town or country, or of both, and what places where anatomy is carried on, situate within such district, every such inspector shall be appointed to superintend, and in what manner every such inspector shall transact the duties of his office.

IV. And be it enacted, that every inspector to be appointed by virtue of this Act shall make a quarterly return to the said secretary of state or chief secretary, as the case may be, of every deceased person's body that during the preceding quarter has been removed for anatomical examination to every separate place in his district where anatomy is carried on; distinguishing the sex, and, as far as is known at the time, the name and age of each person whose body was so removed as aforesaid.

V. And be it enacted, that it shall be lawful for every such inspector to visit and inspect, at any time, any place within his district, notice of which place has been given, as is hereinafter directed, that it is intended there to practise anatomy.

VI. And be it enacted, that it shall be lawful for his Majesty to grant to every such inspector such an annual salary, not exceeding one hundred pounds, for his trouble, and to allow such a sum of money for the expenses of his office as may appear reasonable; such salaries and allowances to be charged on the consolidated fund of the United Kingdom, and to be payable quarterly; and that an annual return of all such salaries and allowances shall be made to parliament.

VII. And be it enacted, that it shall be lawful for any executor or other party having lawful possession of the body of any deceased person, and not being an undertaker or other party intrusted with the body for the purpose only of interment, to permit the body of such deceased person to undergo anatomical examination, unless, to the knowledge of such executor or other party, such person shall have expressed his desire, either in writing at any time during his life, or verbally in the presence of two or more witnesses during the illness whereof he died, that his body after death might not undergo such examination, or unless the surviving husband or wife, or any known relative of the deceased person, shall require the body to be interred without such examination.

VIII. And be it enacted, that if any person, either in writing at any time during his life, or verbally in the presence of two or more witnesses during the illness whereof he died, shall direct that his body after death be examined anatomically, or shall nominate any party by this Act authorized to examine bodies anatomically to make such examination, and if, before the burial of the body of such person, such direction or nomination shall be made known to the party having lawful possession of the dead body, then such last-mentioned party shall direct such examination to be made, and, in case of any such nomination as aforesaid, shall request and permit any party so authorized and nominated as aforesaid to make such examination, unless the deceased person's surviving husband or wife, or nearest known relative, or any one or more of such person s nearest known relatives, being of kin in the same degree, shall require the body to be interred without such examination.

IX. Provided always, and be it enacted, that in no case shall the body of any person be removed for anatomical examination from any place where such person may have died, until after forty-eight hours from the time of such person's decease, nor until after twenty-four hours' notice, to be reckoned from the time of such decease, to the inspector of the district, of the intended removal of the body, or, if no such inspector have been appointed, to some physician, surgeon, or apothecary, residing at or near the place of death, nor unless a certificate stating in what manner such person came by his death shall, previously to the removal of the body, have been signed by the physician, surgeon, or apothecary, who attended such person during the illness whereof he died; or if no such medical man attended such person during such illness, then by some physician, surgeon, or apothecary, who shall be called in after the death of such person to view his body, and who shall state the manner or cause of death according to the best of his knowledge and belief, but who shall not be concerned in examining the body after removal; and that, in case of such removal, such certificate shall be delivered, together with the body, to the party receiving the same for anatomical examination.

X. And be it enacted, that it shall be lawful for any member or fellow of any college of physicians or surgeons, or any graduate or licentiate in medicine, or any person lawfully qualified to practise medicine in any part of the United Kingdom, or any professor, teacher, or student, of anatomy, medicine, or surgery, having a licence from his Majesty's principal secretary of state or chief secretary as aforesaid, to receive or possess for anatomical examination, or to examine anatomically, the body of any person deceased, if permitted or directed so to do by a party who had, at the time of giving such permission or direction, lawful possession of the body, and who had power, in pursuance of the provisions of this Act, to permit or cause the body to be so examined, and provided such certificate as aforesaid were delivered by such party together with the body.]

XI. And be it enacted, that every party, so receiving a body for anatomical examination after removal, shall demand and receive, together with the body, a certificate as aforesaid, and shall, within twenty-four hours next after such removal, transmit to the inspector of the district such certificate, and also a return stating at what day and hour, and from whom, the body was received, the date and place of death, the sex, and (as far as is known at the time) the christian and surname, age, and last place of abode of such person; or, if no such inspector have been appointed, to some physician, surgeon, or apothecary, residing at or near the place to which the body is removed, and shall enter or cause to be entered the aforesaid particulars relating thereto, and a copy of the certificate he received therewith, in a book to be kept by him for that purpose, and shall produce such book whenever required so to do by any inspector so appointed as aforesaid.

XII. And be it enacted, that it shall not be lawful for any party to carry on or teach anatomy at any place, or at any place to receive or possess, for anatomical examination, or examine anatomically, any deceased person's body after the removal of the same, unless such party, or the owner or occupier of such place, or some party by this Act authorized to examine bodies anatomically, shall, at least one week before the receipt or possession of a body for such purpose at such place, have given notice to the said secretary of state or chief secretary, as the case may be, of the place where it is intended to practise anatomy.

XIII. Provided always, and be it enacted, that every such body so removed as aforesaid for the purpose of examination shall, before such removal, be placed in a decent coffin or shell, and be removed therein; and that the party removing the same, or causing the same to be removed as aforesaid, shall make provision that such body, after undergoing anatomical examination, be decently interred in consecrated ground, or in some public burial ground in use for persons of that religious persuasion to which the person whose body was so removed belonged; and that a certificate of the interment of such body shall be transmitted to the inspector of the district within six weeks after the day on which such body was received as aforesaid.

XIV. And be it enacted, that no member or fellow of any college of physicians or surgeons, nor any graduate or licentiate in medicine, nor any person lawfully qualified to practise medicine in any part of the United Kingdom, nor any professor, teacher, or student, of anatomy, medicine, or surgery, having a licence from His Majesty's principal secretary of state or chief secretary as aforesaid, shall be liable to any prosecution, penalty, forfeiture, or punishment, for receiving or having in his possession, for anatomical examination, or for examining anatomically, any dead hnman body, according to the provisions of this Act.

XV. And be it enacted, that nothing in this Act contained shall be constructed to extend to or to prohibit any post-mortem examination of any human body required or directed to be made by any competent legal authority.

XVI. And whereas an Act was passed in the ninth year of the reign of his late Majesty, for consolidating and amending the statutes in England relative to offences against the person, by which latter Act it is enacted, that the body of every person convicted of murder shall, after execution, either be dissected or hung in chains, as to the court which tried the offender shall seem meet; and that the sentence to be pronounced by the court shall express that the body of the offender shall be dissected or hung in chains, whichsoever of the two the court shall order; be it enacted, that so much of the said last-recited Act as authorizes the court, if it shall think fit, to direct that the body of a person convicted of murder shall, after execution, be dissected, be, and the same is hereby repealed; and that every case of conviction of any prisoner for murder, the court before which such prisoner shall have been tried shall direct such prisoner either to be hung in chains or to be buried within the precincts of the prison in which such prisoner shall have been confined after conviction, as to such court shall seem meet; and that the sentence to be pronounced by the court shall express that the body of such prisoner shall be hung in chains, or buried within the precincts of the prison, whichever of the two the court shall order.

XVII. And be it enacted, that if any action or suit shall be commenced or brought against any person for any thing done in pursuance of this Act, the same shall be commenced within six calendar months next after the cause of action accrued; and the defendant in every such action or suit may, at his election, plead the matter specially, or the general issue not guilty, and give this Act and the special matter in evidence at any trial to be had thereupon.

XVIII. And be it enacted, that any person offending against the provisions of this Act, in England or Ireland, shall be deemed and taken to be guilty of a misdemeanour, and, being duly convicted thereof, shall be punished by imprisonment for a term not exceeding three months, or by a fine not exceeding fifty pounds, at the discretion of the court before which he shall be tried; and any person offending against the provisions of this Act in Scotland, shall, upon being duly convicted of such offence, be punished by imprisonment for a term not exceeding three months, or by a fine not exceeding fifty pounds, at the discretion of the court before which he shall be tried.

XIX. And in order to remove doubts as to the meaning of certain words in this Act, be it enacted, that the words "person and party" shall be respectively deemed to include any number of persons, or any society, whether by charter or otherwise; and that the meaning of the aforesaid words shall not be restricted, although the same may be subsequently referred to in the singular number and masculine gender only.

XX. And be it enacted, that this Act shall commence and take effect from and after the 1st day of August in the present year.

XXI. And be it enacted, that this Act may be altered or amended during the present session of parliament.

---

### REGULATIONS IN REGARD TO ANATOMY.

*Office of Inspector of Anatomy:*
5, *Saville row; 30th August,* 1832.

Sir: I am desirous, on being appointed Inspector pursuant to the Act for regulating the Schools of Anatomy, to solicit your co-operation in carrying into execution the enactments of the legislature.

In order to facilitate this object, and to secure uniformity and regularity of proceeding, I beg leave to submit to you a general form of return, as required by the statute, together with an abstract of the duties which appear to me to be imposed by it upon teachers of anatomy. It is unnecessary for me to point out how important it is, for the satisfaction of the public mind, that the natural feelings of human nature should be consulted by a strict attention to the thirteenth section of the Act, which provides for the decent interment of bodies after they have undergone anatomical examination.

I have only to add, that I shall be anxious to receive such advice and information as your knowledge and experience may suggest, and to assure you that any such communications, as well as the returns, shall be considered by me strictly secret and confidential, and shall only be reported to the public authorities, as directed and required by the Act of Parliament.

I am, Sir, your obedient servant,

J. C. SOMERVILLE, M.D.

### *Duties of the Inspector.*

He shall keep an account of all the Schools of Anatomy licensed by the Secretary of State within the district for which he shall be appointed, with the names and residences of the teachers.

He shall make a quarterly return to the secretary of state of the body of every deceased person that during the preceding quarter has been removed for anatomical examination to every separate place in his district where anatomy is carried on; distinguishing the sex, and, as far as is known at the time, the name and age of each person whose body has been so removed as aforesaid.

He shall visit and inspect from time to time every place within his district, of which place notice has been given that it is intended there to practise anatomy; he shall take care that the provisions of the Act of Parliament are enforced, and shall report to the secretary of state any irregularities or offences against the Act which he shall observe.

He shall enter in a register kept for the purpose the returns from each school separately, and shall keep copies of all his returns and reports to the Secretary of State, and shall enter all the correspondence relating to the duties of his office in a letter-book.

*Duties of Teachers of Anatomy, according to the Act for Regulating Schools of Anatomy.*

To obtain a licence according to the provisions of the Act 2d and 3d Will. IV. c. 75.

Upon receiving a body for anatomical examination, to demand and receive with the body a certificate from the physician, surgeon, or apothecary, who attended the person during the illness of which he died, or from some surgeon, physician, or apothecary, who shall have been called in after the death of such person to view the body, stating the manner or cause of the death, and to transmit, within twenty-four hours next after the removal of the body, such certificate to the inspector of the district; and also a return stating at what day and hour, and for whom, the body was received, the date and place of death, the sex, and, as far as is known at the time, the christian and surname, age, and last place of abode, of such person; or, if no such inspector shall have been appointed, to some physician, surgeon, or apothecary, residing at or near the place to which the body is removed; and to enter or cause to be entered the aforesaid particulars, and a copy of the certificate, in a book to be kept by him for that purpose, and to produce such book whenever required by the inspector.

To give notice to the Secretary of State of every place where it is intended to practise anatomy, at least one week before the first receipt or possession of a dead body for such purpose at such place.

To take care that every body removed for examination shall be removed in a decent and fitting manner, and to make provision that such body, after undergoing anatomical examination, be decently interred, according to the thirteenth clause of the said Act; and that a certificate of the interment of such body be transmitted to the inspector of the district within six weeks after the day of the reception of such body.

*Form of Application.*

The party applying for a licence to practise anatomy must make a written application to the Secretary of State, signed by him with his christian and surname at length, in which application he must state, he applies for a licence to practise anatomy, in pursuance of the Act passed for regulating Schools of Anatomy. He must state also the place of his residence, and the place where he is about to carry on the practice of anatomy, and must further state, that he is, according as the case may be, [a fellow or member of a college of physicians or surgeons], or [a graduate or licentiate in medicine,] or [a person lawfully qualified to practise medicine in England, Scotland, or Ireland,] or [a professor or teacher of anatomy, medicine, or surgery,] or [a student attending a school of anatomy].

This application is to be countersigned by two justices of the peace acting for the county, city, or borough, wherein the party applying resides, and so describing themselves, certifying the place of residence of the party, and also the place where they believe him to be about to carry on the practice of anatomy.

*Confidential Return from the School of Anatomy at*
*as required by the 2d and 3d* William IV. cap. 75.

| The Christian Name and the Surname of the Deceased. | Age and Sex. | Last Place of Abode of deceased Person. | Date and Place of Death. | Time of Removal. | Day and Hour at which the Body was received, and from whom received. | Observations. |
|---|---|---|---|---|---|---|
| | | | | | | |

(Signed) — — Teacher.

### MONTHLY LIST OF MEDICAL BOOKS.

### METEOROLOGICAL JOURNAL,

By Messrs. Harris and Co. Mathematical Instrument Makers, 50, High Holborn.

| | Rain gauge. | Moon. | Thermometer. | | | Barometer. | | De Luc's Hygrometer. | | Winds. | | Atmospheric Variation. | | |
|---|---|---|---|---|---|---|---|---|---|---|---|---|---|---|
| *Aug.* | | | 9a.m. | max. | min. | 9a.m. | 10p.m. | 9a.m. | 10p.m. | 9 a.m. | 10p.m. | 9 a. m. | 2 p.m. | 10 p. m. |
| 20 | | ☾ | 62 | 69 | 63 | 29.78 | 29.72 | 53 | 53 | W | SW | fine | fine | cloudy |
| 21 | | | 63 | 67 | 58 | .62 | .57 | 53 | 54 | WSW | SW | cloudy | rain | |
| 22 | | | 64 | 70 | 57 | .50 | .57 | 57 | 58 | WSW | WSW | | cloudy | fine |
| 23 | | | 61 | 68 | 54 | .67 | .68 | 55 | 54 | SW | WSW | fine | fine | |
| 24 | .32 | | 64 | 67 | 58 | .80 | .80 | 54 | 54 | W | SW | | | cloudy |
| 25 | | ● | 60 | 65 | 48 | .59 | .53 | 55 | 51 | SSW | S W | cloudy | | |
| 26 | | | 54 | 59 | 50 | .56 | .50 | 51 | 52 | W | NW | fine | cloudy | fine |
| 27 | | | 59 | 65 | 57 | .51 | .36 | 53 | 60 | SSW | SW | cloudy | rain | cloudy |
| 28 | | | 60 | 64 | 53 | .02 | .06 | 57 | 58 | S | NW | cloudy | cloudy | |
| 29 | .33 | | 55 | 59 | 55 | .12 | .19 | 58 | 59 | W | WNW | | rain | Showery |
| 30 | | | 60 | 60 | 52 | .30 | .42 | 62 | 62 | WNW | NW | rain | rain | rain |
| 31 | .33 | | 60 | 62 | 65 | .53 | .49 | 61 | 61 | WSW | SSW | fine | fine | cloudy |
| *Sept.* | | | | | | | | | | | | | | |
| 1 | .09 | | 60 | 63 | 53 | .41 | .50 | 60 | 56 | WSW | WNW | | cloudy | |
| 2 | .20 | ☽ | 58 | 64 | 53 | .63 | .88 | 56 | 55 | WNW | WNW | | fine | |
| 3 | | | 60 | 65 | 54 | .94 | .97 | 55 | 54 | WNW | N | | | fine |
| 4 | | | 57 | 62 | 57 | 30.03 | .93 | 54 | 54 | N | ESE | | | |
| 5 | .03 | | 60 | 63 | 54 | 29.85 | .82 | 54 | 53 | ENE | NE | | | |
| 6 | | | 62 | 63 | 57 | .73 | .69 | 53 | 57 | E | E | | | rain |
| 7 | | | 61 | 62 | 56 | .64 | .70 | 59 | 61 | WSW | W | cloudy | | fine |
| 8 | | | 60 | 67 | 55 | .80 | .80 | 60 | 55 | W | W | fine | | |
| 9 | | | 60 | 64 | 54 | .78 | .67 | 57 | 56 | WSW | SW | | | |
| 10 | | ○ | 60 | 66 | 53 | .54 | .67 | 56 | 55 | SW | W | rain | | |
| 11 | | [illegible] | 61 | 63 | 50 | .86 | .99 | 55 | 53 | NW | WNW | fine | | |
| 12 | | | 58 | 60 | 57 | 30.06 | .98 | 52 | 54 | W | WSW | | | |
| 13 | | | 60 | 62 | 51 | 29.82 | .78 | 57 | 56 | WSW | W | rain | | cloudy |
| 14 | | | 57 | 59 | 54 | .65 | .65 | 54 | 53 | W | WNW | fine | | fine |
| 15 | .20 | | 59 | 62 | 49 | .74 | .91 | 53 | 51 | NW | NW | | cloudy | |
| 16 | | | 57 | 63 | 52 | .97 | 30.01 | 54 | 53 | NW | SW | | | |
| 17 | | ☾ | 60 | 64 | 57 | 30.05 | 29.96 | 53 | 54 | WSW | SW | cloudy | fine | |
| 18 | | | 62 | 65 | 47 | 29.74 | .87 | 54 | 52 | SW | NNW | | cloudy | |
| 19 | | | 53 | 54 | 44 | 30.07 | 30.22 | 52 | 54 | N | NNE | fine | fine | |

The quantity of Rain fallen in August, was 2 inches and 9.100ths.

